# Multiple cerebral infarctions in a patient with idiopathic thrombocytopenic purpura

**Published:** 2016-07-06

**Authors:** Masatoshi Yunoki, Kenta Suzuki, Atsuhito Uneda, Shuichi Okubo, Koji Hirashita, Kimihiro Yoshino

**Affiliations:** ^1^ Department of Neurosurgery, Kagawa Rosai Hospital, Kagawa, Japan

**Keywords:** Cerebral Infarction, Idiopathic Thrombocytopenic Purpura, Neuroimaging

Idiopathic thrombocytopenic purpura (ITP) is an autoimmune condition with detectable antibodies against several platelet surface antigens. The diagnostic criteria of ITP include isolated thrombocytopenia, normal bone marrow, and absence of other causes of thrombocytopenia. This condition is characterized by minor and serious bleeding complications, but it is rarely accompanied by thrombosis.^1^ We describe a patient with ITP that was not previously diagnosed, who developed cerebral infarction a few days after undergoing platelet transfusion and initiating steroid therapy. 

Our patient was a 31-year-old woman, with no prior medical history, who presented at the gynecology department of our hospital with heavy menstrual bleeding as her chief complaint. She was referred to hematology department and admitted as the initial laboratory evaluation showed microcytic anemia [red blood cell (RBC) count: 302 × 10^4^/µl, hemoglobin: 7.8 g/dl, hematocrit: 23.3%] and a platelet count of 0.6 × 10^4^/µl. Extensive blood tests were performed including platelet-associated IgG, lupus anticoagulant, antinuclear factor, anti-cardiolipin antibody (ACA), and anti-Helicobacter pylori (H. pylori) immunoglobulin G, and all the results were either normal or negative. 

She underwent platelet transfusion (20 IU in a single administration) and steroid therapy (methylprednisolone 1000 mg/day for 5 days and 50 mg of prednisolone thereafter). After treatment, the platelet count on the second day of hospitalization increased to 13.2 × 10^4^/µl and remained within normal levels thereafter. The patient, however, gradually became disoriented. A brain computed tomography scan 3 days after admission showed multiple low-density areas in both frontal lobes (Figure 1, A and B). Thus, the patient was referred to neurosurgery department. 

The bifrontal lesion was found to be acute cerebral infarction using diffusion-weighted magnetic resonance (MR) imaging (Figure 1, C and D). The MR angiography revealed stenosis in bilateral distal internal carotid arteries and middle cerebral arteries (Figure 1, E); therefore, a cerebral angiography was performed. In the cerebral angiography, mild stenosis of the bilateral distal internal carotid artery (ICA) and stenosis of the middle cerebral artery (MCA) were confirmed. 

**Figure 1:**
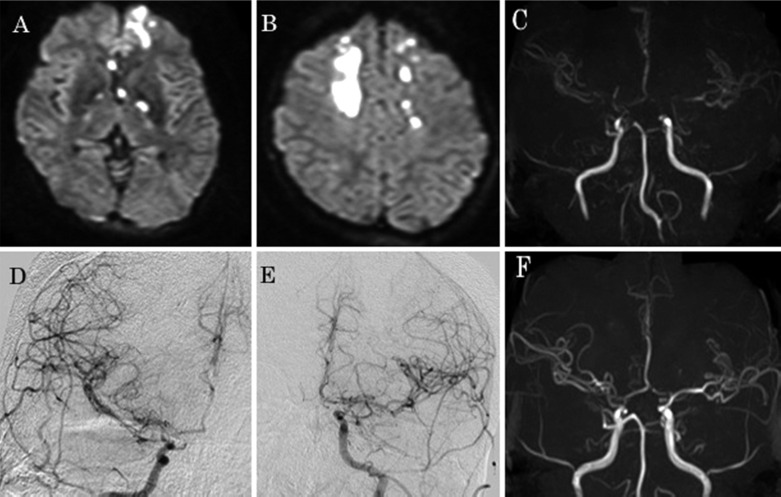
Magnetic resonance (MR) assessments for the patient

Diffusion-weighted MR images show acute ischemic lesions (A, B). MR angiography at the time of presentation to neurosurgery revealed distal and bilateral stenosis of the internal carotid artery (ICA) and middle cerebral artery (MCA) (C). On the left (D) and right (E) cerebral angiography, moderate stenosis was identified in bilateral distal ICA and MCA. In MR angiography 7 weeks after admission, stenosis of bilateral ICA and MCA considerably improved (F).

Antiplatelet therapy for the newly acquired stroke was initiated considering the patient’s platelet count remained within the normal range with steroid therapy. Symptoms gradually improved, and she was discharged from the hospital 3 weeks after admission. In a follow-up MR angiography taken 7 weeks after admission, the bilateral ICA stenosis had improved considerably (Figure 1, F).

Generally, ITP is not a life-threatening disease; therefore, treatment may not be needed in mild cases. Treatment is usually initiated when the patient’s platelet count is less than 2.0 × 10^4^/µl. A causal link between H. pylori infection and ITP has been suggested in previous clinical studies, showing a platelet count response in approximately 50% of patients following H. pylori eradication.^2^ A new treatment guideline, therefore, has been proposed in which the presence of H. pylori infection is confirmed first and the eradication therapy is administered in positive cases.^2^ As H. pylori infection was not observed in our patient, steroid treatment was used as the backbone of therapy. Additionally, platelet transfusion was administered during the critical stage to rapidly increase the platelet count, which was less than 1.0 × 10^4^/µl and was associated with heavy menstrual bleeding. 

In the literature, ischemic complications are reported after the treatment for ITP. Moreover, a previous report described cases of myocardial ischemia after a platelet count increase, resulting from intravenous immunoglobulin therapy or splenectomy.^3^ When the platelet count and viscosity of the blood increase as a result of treatment, the risk of thrombotic complications may also increase. The risk of ischemic complications should be considered when treating these patients with thrombocytopenia. 

In contrast, some cases have been reported in which ischemic complications occurred in patients with ITP even when platelet count was extremely low.^1^ Antiplatelet antibodies induce complement-mediated platelet fragmentation and induce platelet microparticles (PMPs). These PMPs play an important role in the hemostasis of patients with thrombocytopenia. A high level of hemostatically active PMPs can be thrombogenic in certain clinical settings.^4^ Patients with ITP often present with higher PMP levels, which may lead to an increase in thrombotic events.^4^

We used antiplatelet therapy because the patient’s platelet count remained within normal levels with steroid therapy, and a successful outcome was achieved. However, the management of ischemic stroke in patients with ITP remains controversial. Therapy for the ischemic stroke should be individualized according to the presumed pathophysiologic mechanism of stroke, comorbidity, and estimated risk of hemorrhagic complications.

As far as we have been able to ascertain from the literature, this case is the first to report improved arterial stenosis after antiplatelet therapy. Because of this change in MR arteriography findings, reversible cerebral vasoconstriction syndrome, which is reported in cases of thrombotic thrombocytopenic purpura,^5^ should be included in the differential diagnosis. Reversible cerebral vasoconstriction syndrome is a cerebrovascular disorder associated with multifocal arterial constriction and dilation. Its primary clinical presentation is characterized by recurrent and severe thunderclap headaches over 1-3 weeks, often accompanied by nausea, vomiting, photophobia, confusion, and blurred vision.^5^ As these symptoms were not present in our case, we speculate that the change in the MR arteriography finding of our case illustrates the thrombolysis occurring gradually in major intracranial arteries after antiplatelet therapy.

This case showed a mutually exclusive relationship between ITP and ischemic stroke. In patients with ITP, the risk of thrombotic complications should be considered, especially when treatment is given to rapidly increase the platelet count.

## Notes:

**How to cite this article:** Yunoki M, Suzuki K, Uneda A, Okubo S, Hirashita K, Yoshino K. Multiple cerebral infarctions in a patient with idiopathic thrombocytopenic purpura. Iran J Neurol 2016; 15(3): 177-9.
